# Efficacy of lenvatinib *versus* sorafenib in the primary treatment of advanced hepatocellular carcinoma: A meta‐analysis

**DOI:** 10.1002/jgh3.12999

**Published:** 2023-12-13

**Authors:** Vikash Jaiswal, Maha Hameed, Sidra Naz, Poulami Roy, Novonil Deb, Janta Ukrani, Gautham Varun Krishna Mohan, Amira M Taha, Helen Huang, Vikash Kumar, Bhavyakumar Vachhani, Abdelrahman M Attia, Supti D Nath, Mostafa A Solimn, Dattatreya Mukherjee

**Affiliations:** ^1^ Department of Research Larkin Community Hospital South Miami Florida USA; ^2^ Department of Internal Medicine Florida State University, Sarasota Memorial Hospital Sarasota Florida USA; ^3^ Department of Gastroenterology The University of Texas, MD Anderson Cancer Center Houston Texas USA; ^4^ North Bengal Medical College and Hospital West Bengal India; ^5^ Department of Internal Medicine Mather Hospital, Northwell Health Port Jeffersona New York USA; ^6^ Department of Medicine Tirunelveli Medical College Tirunelveli India; ^7^ Faculty of medicine Fayoum University Fayoum Egypt; ^8^ Royal College of Surgeons in Ireland University of Medicine and Health Science Dublin Ireland; ^9^ Department of Internal Medicine The Brooklyn Hospital Center New York New York USA; ^10^ Faculty of medicine Cairo University Cairo Egypt; ^11^ Department of Medicine Johns Hopkins University Baltimore Maryland USA; ^12^ Department of Medicine Raiganj Government Medical College and Hospital West Bengal India

**Keywords:** hepatocellular carcinoma, lenvatinib, mortality, sorafenib

## Abstract

**Background and Aim:**

Molecular‐targeted agents such as lenvatinib and sorafenib have been approved to treat hepatocellular carcinoma (HCC). However, the choice between these two agents in the primary treatment for advanced HCC is still under debate with conflicting results. We sought to evaluate the efficacy of lenvatinib and sorafenib in patients with HCC.

**Methods:**

We performed a systematic literature search using PubMed, Embase, and Scopus for relevant articles from inception until February 10, 2023. The primary outcome of this meta‐analysis was overall survival (OS). The secondary outcomes were progression‐free survival (PFS), time to progression, objective response rate (ORR), and disease control rate (DCR).

**Results:**

A total of 13 studies with 3705 patients (1635 on lenvatinib and 2070 on sorafenib) were included in our analysis. The mean age of the patients in both groups was comparable (66.81 *vs* 65.9 years). Pooled analysis of primary outcomes showed that, compared with sorafenib, lenvatinib was associated with significantly better OS in patients treated with these drugs (HR 0.82, 95% CI: 0.69–0.97, *P* = 0.02). Pooled analysis also showed that PFS (HR 0.67, 95% CI: 0.57–0.78, *P* < 0.00001) and time to progression (HR 0.49, 95% CI: 0.31–0.79; *P* = 0.004) were significantly better in the lenvatinib group compared to the sorafenib group. It also showed that the lenvatinib group had significantly better ORR (odds ratio [OR] 5.43, 95% CI: 3.71–7.97; *P* < 0.00001) and DCR (OR 2.35, 95% CI: 1.75–3.16; *P* < 00001) than the sorafenib group.

**Conclusion:**

Our study shows that lenvatinib is superior to sorafenib regarding OS and PFS in patients with advanced HCC.

## Graphical abstract

Our study shows that lenvatinib was superior to sorafenib in overall survival and progression‐free survival in advanced hepatocellular carcinoma patients.

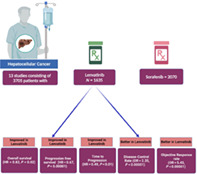

## Introduction

Hepatocellular carcinoma (HCC) is the most prevalent primary hepatic malignancy contributing substantially to global cancer‐related mortality. HCC cases have been on the rise, mainly because of their aggressive nature and limited treatment modalities.^1^ HCC is the predominant subtype of hepatic malignancy globally, constituting approximately 75% of overall cases.^2^ Irrespective of geographical location, HCC is usually associated with a poor prognosis.^3^ As of 2018, the approximate annual incidence of HCC was 9.3 cases per 100 000 individuals, with a corresponding mortality rate of 8.5 cases per 100 000 person‐years.^4^ These figures lend support to the notion that HCC is associated with an unfavorable prognosis.

Because of the asymptomatic presentation of HCC in the initial stages, a considerable proportion of HCC cases go unnoticed and diagnosed later in advanced or unresectable stages, which culminates in irreversible pathological states that defy any attempts at remediation.^5^ In cases that do not present evidence of advanced liver fibrosis and portal hypertension, surgical resection of the tumor is regarded as the optimal course of treatment. However, it is noteworthy that liver surgery in patients with chronic liver disease is associated with an elevated risk of hepatic failure, particularly in the case of extended resections.^6^ Implementing efficient therapeutic interventions is imperative to impede the disease's swift progression, thus ultimately reducing fatality.

Over the past few decades, drug development endeavors for HCC have experienced major setbacks, characterized by four global Phase III trials (namely sunitinib, brivanib, linifanib, and erlotinib plus sorafenib) that yielded unsatisfactory results.^7^, ^8^, ^9^, ^10^ Specifically, these trials failed to demonstrate non‐inferiority or superiority compared to sorafenib regarding overall survival (OS) as a first‐line treatment for HCC. Sorafenib and lenvatinib are the widely adopted first‐line systemic treatments for advanced HCC.^11^ However, there is a shortage of literature pertaining to the potential benefits that lenvatinib may offer over sorafenib in terms of efficacy, depending on the specific needs of the patient cohort. Through synthesis and analysis of available clinical data, our systematic review and meta‐analysis aims to provide an objective and evidence‐based evaluation of these two chemotherapeutic agents' relative merits and demerits, which could aid in clinical decision making and improve disease outcomes.

## Materials and methods

This meta‐analysis was conducted and reported following the Cochrane and PRISMA (Preferred Reporting Items for Systematic review and Meta‐Analysis) 2020 guidelines and performed according to established methods, as described previously.^7^, ^8^, ^9^ The prespecified study protocol has been registered in PROSPERO (CRD42023400480).

### Search strategy

We conducted a systematic literature search in PubMed, Embase, and Scopus using predefined MESH terms by using “AND” and “OR.” The following search terms were used: “Hepatocellular Carcinoma” OR “HCC” OR “Liver Cancer” AND “Lenvatinib” AND “Sorafenib.” We queried databases from their search inception until February 10, 2023 without any restrictions on the language of publication. The search strategies are listed in Table S1.

All the studies were carefully screened and exported to the Mendeley Reference Manager used to handle searched citations. A manual cross‐checking was carried out to remove any duplicates. Two reviewers (V.J. and S.N.) reviewed the papers based on their titles and abstracts. Any disputes regarding the inclusion of studies were arbitrated by another author (A.J.).

### Eligibility criteria

We included studies with adult patients aged ≥18 years. There was no restrictions on the language of publication. All randomized controlled trials (RCTs) as well as prospective and retrospective cohort studies were considered eligible for inclusion. It was decided to include studies with two arms so a that comparison could be made, with one arm consisting of patients on lenvatinib and the other consisting of patients on sorafenib. Those studies that compare patients with varying baseline characteristics and pathologies along with a head‐on comparison with data for efficacy outcomes were also eligible.

Studies performed on animals, reviews, case reports, case series, studies on patients <18 years, studies with a single arm or without HCC, and studies without outcomes of interest were excluded from the review.

### Clinical outcomes

The primary outcome of this meta‐analysis was OS. The secondary outcomes were progression‐free survival (PFS), time to progression, objective response rate (ORR), and disease control rate (DCR).

### Data extraction and quality assessment

Two authors (V.A. and D.M.) extracted the following data: study type, author, study location, study follow‐up duration, patient characteristics (number, age, gender, and comorbidities), and primary and secondary outcomes. We used the reported estimates when reported in the form of hazard ratios (HRs). If different estimates were available, we opted for HR with the most adjusted effect measure or propensity‐score‐matched data where available. Two investigators (V.J. and S.N.) independently appraised the potential risk of bias using the Newcastle‐Ottawa scale (NOS) for observational studies^12^ and Robbin's risk‐of‐bias tools for RCTs.^13^ We then classified studies as of low, moderate, or high quality based on the scores after evaluation.

Statistical analyses were performed by calculating the HR for time‐to‐event outcomes, using the random effects model, with a test for overall effect reported as the *Z*‐value, 95% confidence interval (CI), and the probability value (*P*‐value). Statistical significance was met if 95% CI did not cross “1” and *P* < 0.05. The heterogeneity among studies was assessed by Higgins's statistical model with *I* ^2^ values. As a guide, *I* ^2^ < 25% indicated low heterogeneity, 25–50% moderate heterogeneity, and >50% high heterogeneity.^14^ Publication bias was assessed for primary outcomes with at least five studies using the graphical presentation of funnel plot asymmetry.^15^ All statistical analyses were performed using Review Manager software (RevMan) Version 5.4.

## Results

### Baseline characteristics of patients in included studies

The initial search yielded 1332 articles, from which 253 duplicates were removed and 1034 were excluded after title and abstract screening. The full‐text review was performed on the remaining 45 studies, of which 32 studies were excluded from the final review and analysis for the following reasons: lack of appropriate comparison arm, wrong population, overlapped population, non‐HCC patients, or lack of outcome of interest. Finally, a total of 13 studies met the eligibility criteria and were included in the meta‐analysis.^10^, ^11^, ^12^, ^13^, ^14^, ^15^, ^16^, ^17^, ^18^, ^19^, ^20^, ^21^, ^22^ The Preferred Reporting Items for Systematic Reviews and Meta‐Analyses (PRISMA) flow diagram is shown in Figure S1.

In summary, 13 studies with 3705 patients were included in the final analysis, of which 1635 patients were on lenvatinib and 2070 patients were on sorafenib. The mean age of the patients in both groups was comparable (66.81 ± 13.6 *vs* 65.9 ± 14.9 years). The number of males in the lenvatinib and sorafenib groups was 43.5% and 56.5%, respectively. Most of the patients had Barcelona Clinic liver cancer (BCLC) stage C, and most patients were in Child–Pugh stage A rather than stage B (Table 1). Besides, the the NOS score ranged from 7 to 9, indicating a high quality of all 12 included cohort studies, while the risk of bias for the only RCT was low (Tables S1 and S2).

**Table 1:** Baseline demographics, comorbidities, and characteristics of studies included in the meta‐analysis

Author	Sample	Design	Age	Male	ECOG 0/1	Hepatitis C/B	BCLC stage B/C	Child–Pugh class A/B
Kudo *et al*.	478/476	RCT	61.3/61.2	405/401	304:174/301:175	91:251/126:228	104:374/92:384	475:3/471:5
Kuzuya *et al*.	13/28	Cohort	70/67	11/21	12:1/18:10	2:2/8:8	0:13/0:28	13:0/28/0
Nakano *et al*.	146/146	Cohort	72/72	125/121	—	77:25/81:24	79:67/81:65	134:12/137:9
Tomonari *et al*.	52/52	Cohort	70/71	36/35	38:14/37:15	18:15/19:10	27:25/29:23	52:0/52/0
Choi *et al*.	44/88	Cohort	58/58	40/80	32:12/55:33	‐	4:39/8:77	29:13/63:19
Burgio *et al*.	144/144	Cohort	—	111/119	114:30/114:30	67:22/70:31	36:108/36:108	137:7/134:10
Rimini *et al*.	92/92	Cohort	—	75/81	70:22/65:27	38:18/41:15	36:56/36:56	87:5/85:7
Lee *et al*.	22/44	Cohort	63.95/63.77	18/36	—	6:12/13:24	0:22/0:44	22:0/44:0
Kuo *et al*.	70/140	Cohort	65/65.7	50/100	—	22:36/34:75	14:56/25:115	68:2/138:2
Casadei *et al*.	385/555	Cohort	72.1/62.6	303/485	—	—	−/175:483	339:46/512:43
Park *et al*.	34/60	Cohort	62/65	29/52	—	170:52/169:236	1:29/4:52	30/56
Fukushima *et al*	110/110	Cohort	73/72	91/94	—	36:28/44:27	59:49/86:24	86:24/85:25
Terashima *et al*.	45/135	Cohort	70/69	33/96	36:8/106:22	22:11/59:34	‐	39:6/11421

All data are arranged in the order lenvatinib/sorafenib.

### Meta‐analysis of primary and secondary outcomes

**Figure 1:**
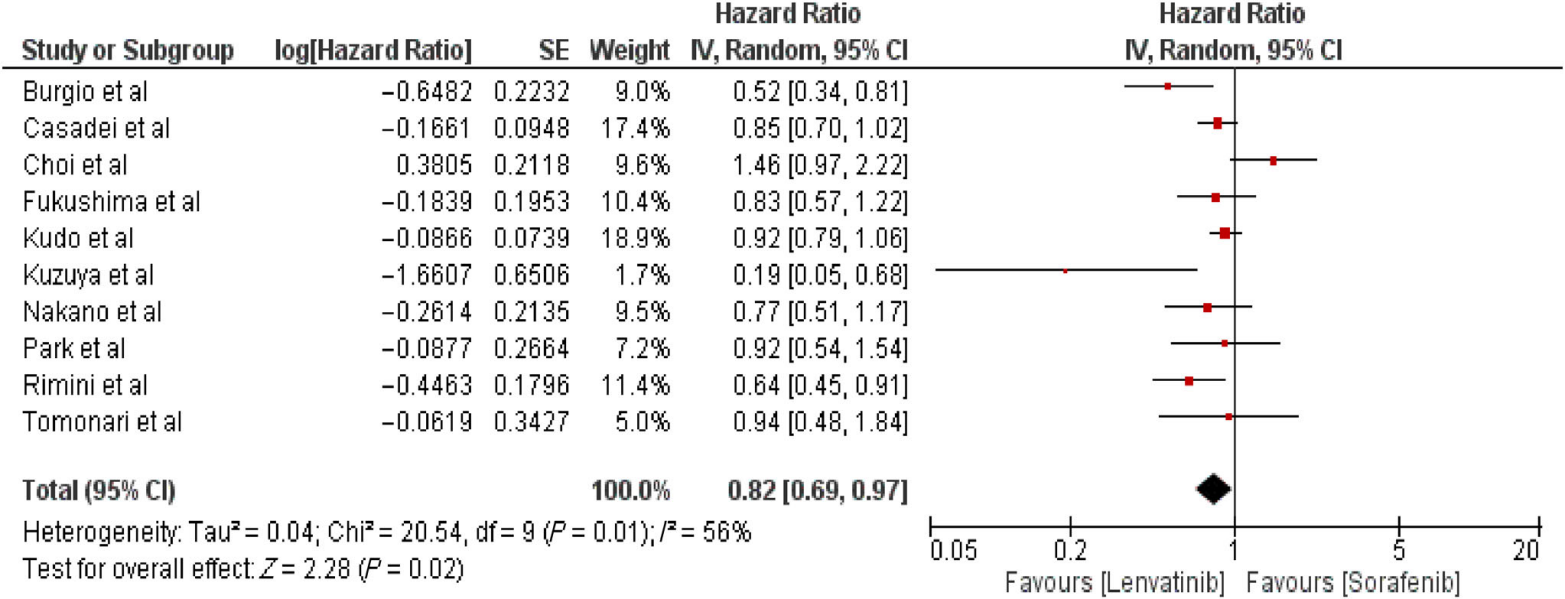
Meta‐analysis showing the forest plot of primary outcome: Overall survival.

Pooled analysis of the primary outcome showed that compared with the patients in the sorafenib group, those in the lenvatinib group were associated with significantly improved OS (HR 0.82, 95% CI: 0.69–0.97; *P* = 0.02, *I* ^2^ = 56%) (Fig. 1).

**Figure 2:**
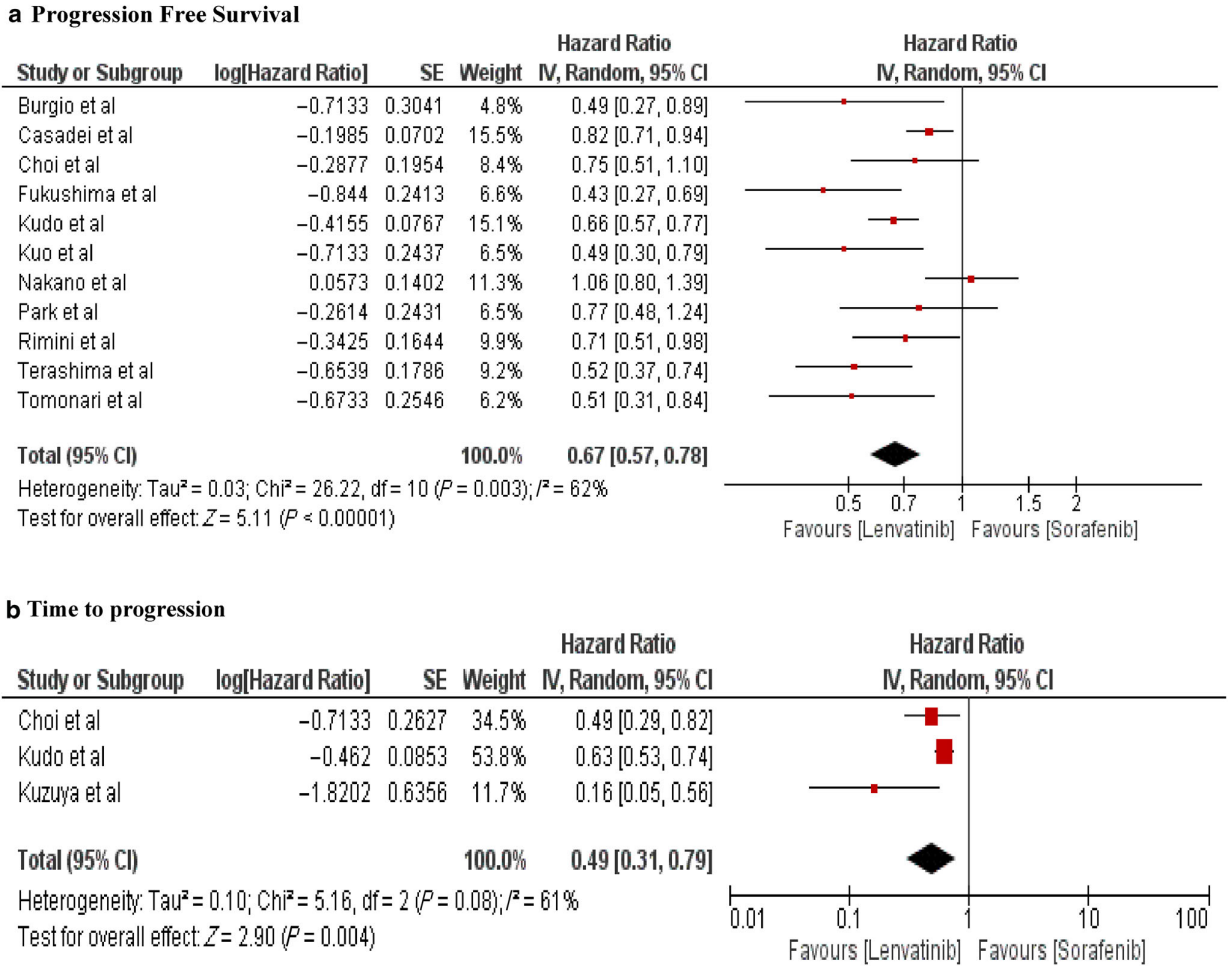
Forest plot of secondary outcomes: (a) progression‐free survival, and (b) time to progression.

Pooled analysis of secondary outcomes showed that patients in the lenvatinib group were associated with significantly better PFS (HR 0.67, 95% CI: 0.57–0.78; *P* < 00001, *I* ^2^ = 62%) and time to progression (HR 0.49, 95% CI: 0.31–0.79; *P* = 0.004, *I* ^2^ = 61%) compared with those in the sorafenib group (Fig. 2a,b).

**Figure 3:**
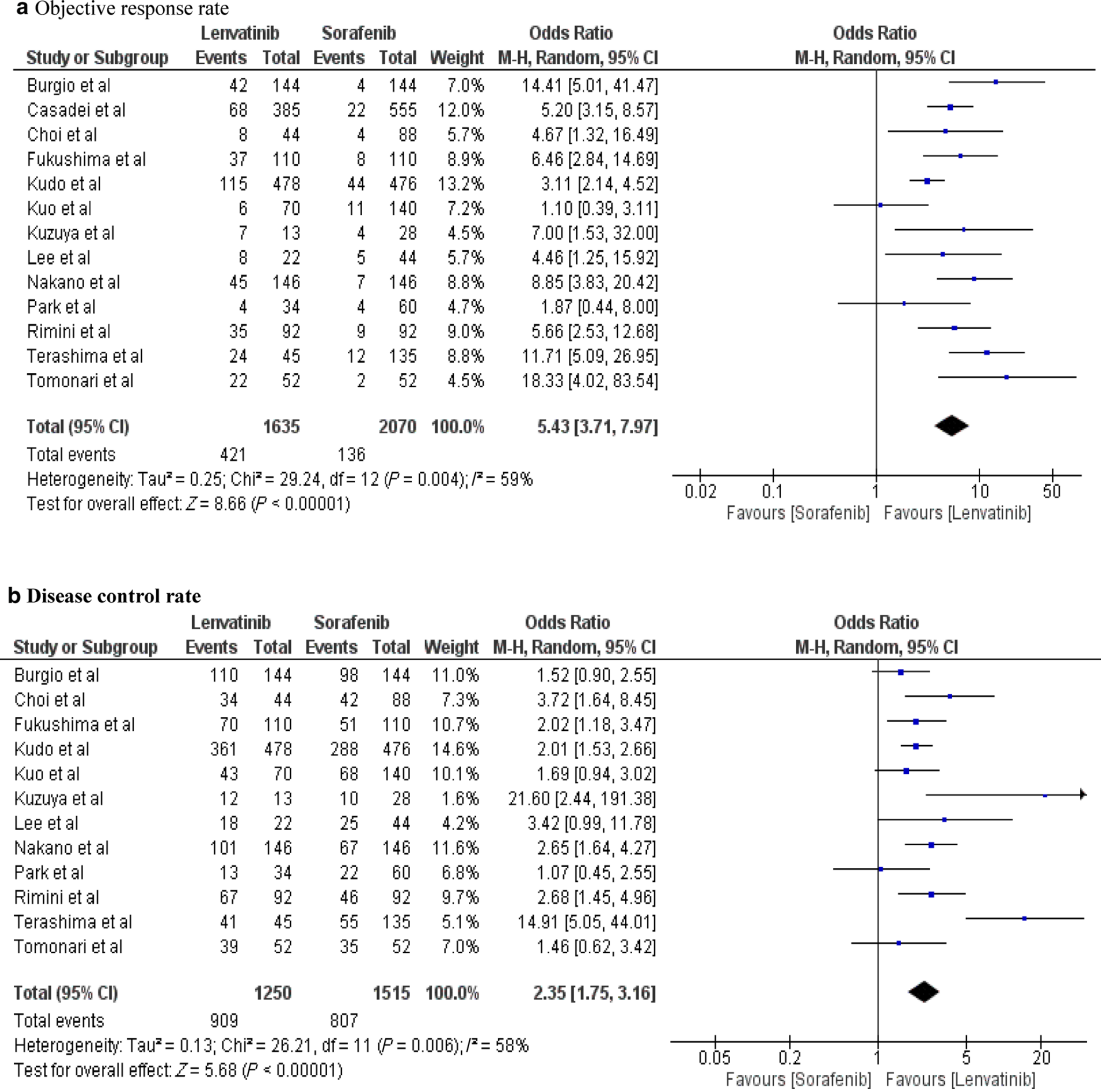
Forest plot of secondary outcomes: (a) objective response rate, and (b) disease control rate.

ORR and DCR were used to evaluate tumor treatment response. Pooled analysis showed that the lenvatinib group had significantly better ORR (OR 5.43, 95% CI: 3.71–7.97; *P* < 0.00001, *I* ^2^ = 59) and DCR (OR 2.35, 95% CI: 1.75–3.16; *P* < 00001, *I* ^2^ = 58%) than the sorafenib group (Fig. 3a,b).

### Subgroup analysis

Studies with a sample size >200 showed a significant difference between the groups, favoring lenvatinib over sorafenib in OS (HR 0.83, 95% CI: 0.71–0.95], *P* = 0.009, *I* ^2^ = 34%), whereas studies with a sample size <200 did not show any significant difference (HR 0.81, 95% CI: 0.51–1.30; *P* = 0.38, *I* ^2^ = 72%) (Fig. S2). However, there was no statistically significant difference in effect between the two subgroups (*P* = 0.95) (Fig. S2).

Regarding PFS, the lenvatinib group experienced significantly better outcomes in studies with sample sizes <200 (HR 0.65, 95% CI: 0.54–0.77; *P* < 00001, *I* ^2^ = 0%) and >200 (HR 0.68, 95% CI: 0.54–0.85, *P* = 0.0007, *I* ^2^ = 76%). There was no significant difference in effect between the subgroups (*P* = 0.74) (Fig. S3).

In terms of time to progression, subgroup analysis showed that studies with both sample sizes (<200 [HR 0.33, 95% CI: 0.12–0.93]; *P* = 0.04, *I* ^2^ = 61%) and >200) showed a significant difference, favoring lenvatinib over sorafenib (HR 0.63, 95% CI: 0.53–0.74; *P* < 00001). There was no statistically significant difference in effect between the two subgroups (*P* = 0.23) (Fig. S4).

For ORR, patients in studies with both sample sizes ([<200 (HR 6.49, 95% CI: 4.02–10.48; *P* < 00001, *I* ^2^ = 17%] and >200 [HR 4.86, 95% CI: 2.80–8.44; *P* < 00001, *I* ^2^ = 74%]) experienced a significant difference, favoring lenvatinib over sorafenib, with no statistically significant difference in effect between the subgroups (*P* = 0.44). In terms of DCR, subgroup analysis showed better outcomes in the lenvatinib group for both sample sizes (<200 [HR 3.33; 95% CI: 1.70–6.54, *P* = 0.0005, *I* ^2^ = 71%] and >200 [HR 1.99, 95% CI: 1.64–2.41, *P* < 00001, *I* ^2^ = 0%]). There was no significant difference between the two subgroups (*P* = 0.15) (Figs. S5 and S6).

To detect publication bias, we used a funnel plot, which appeared symmetrical with no evidence of bias (Fig. S7).

## Discussion

**Figure 4:**
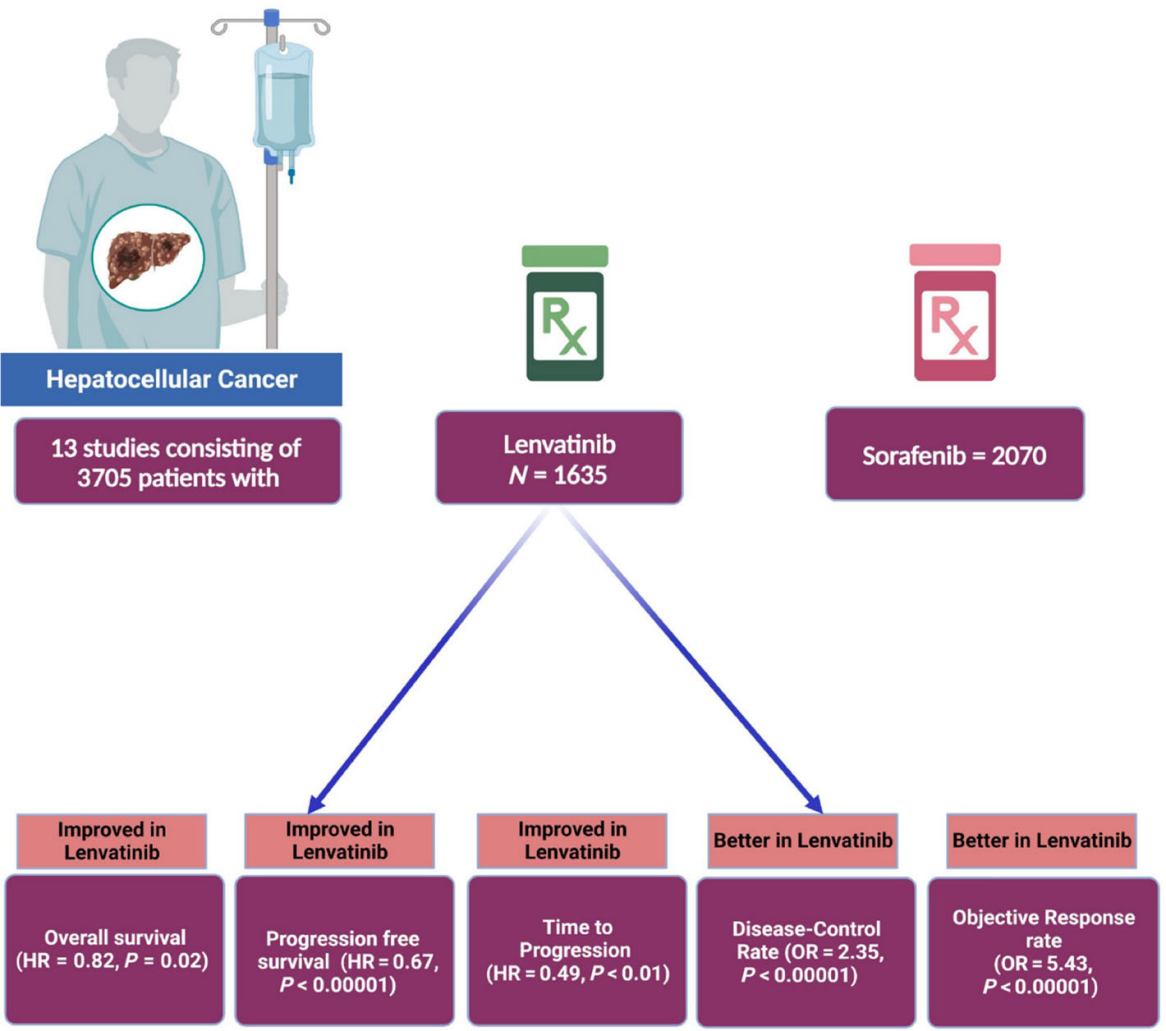
Central illustration showing the clinical findings among hepatocellular carcinoma patients on lenvatinib and sorafenib.

The findings of this meta‐analysis show significant improvement in OS in advanced HCC patients treated with lenvatinib. PFS, ORR, and DCR were significantly higher in the lenvatinib group than the sorafenib group, thus demonstrating the beneficial effects of therapy with lenvatinib (Fig. 4).

### Mechanism of action

**Figure 5:**
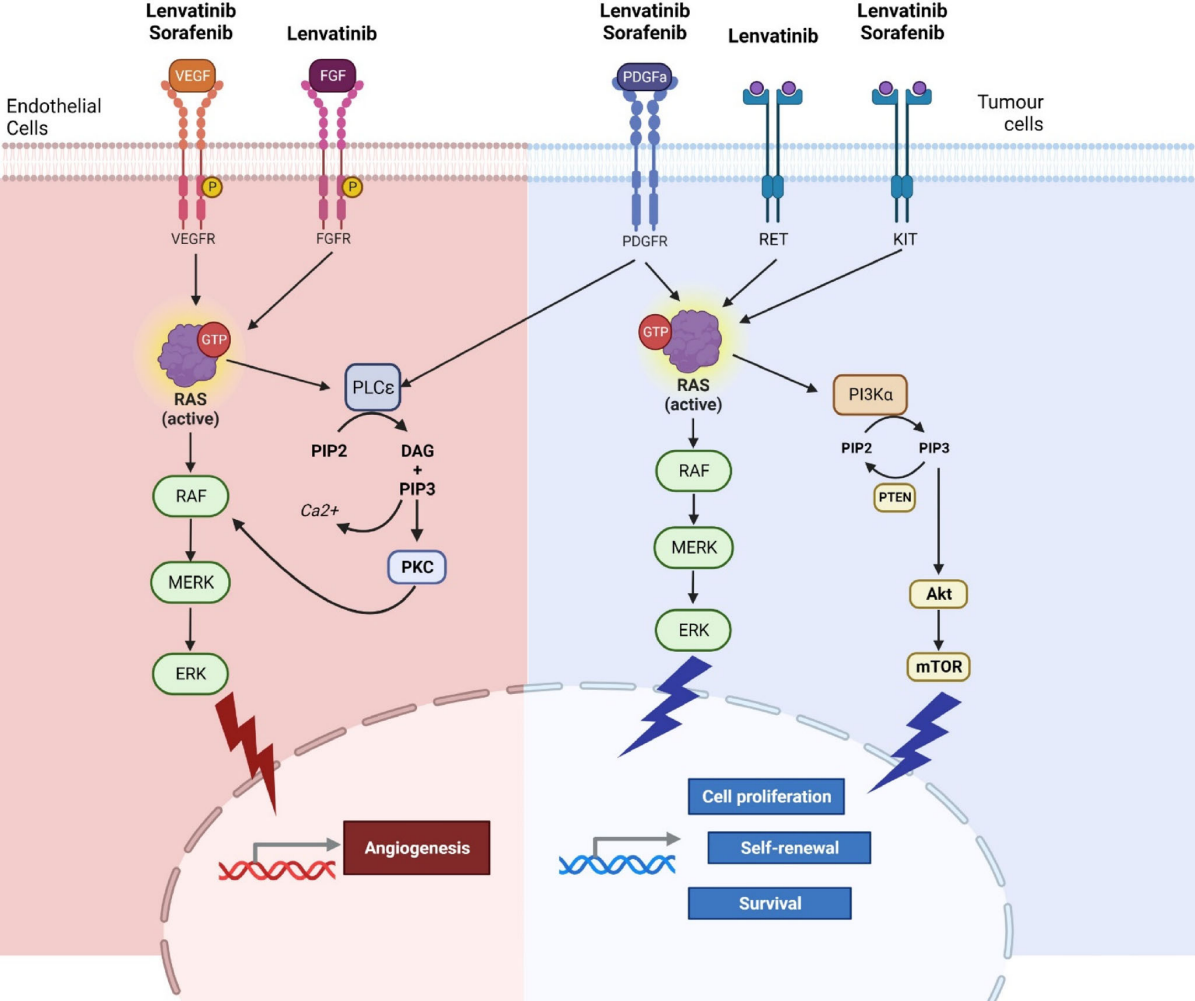
Mechanism of action for lenvatinib and sorafenib on tumor angiogenesis, cell proliferation, and survival in hepatocellular carcinoma.

Lenvatinib is an FDA‐approved, multitargeted tyrosine kinase inhibitor (TKI) that is administered orally either as monotherapy for unresectable or advanced HCC), or in conjunction with additional treatments for other malignancies because of its potent anti‐angiogenic and antitumor properties.^23^, ^24^ Lenvatinib exhibits a pharmacokinetic profile characterized by peak plasma concentrations occurring 2–4 h after dosing, a large volume of distribution, and hepatic metabolism via the cytochrome P450 (CYP)3A4 enzyme system. The drug's half‐life is relatively long at 28 h.^25^ Fecal excretion accounts for about two‐thirds of total elimination, while approximately one‐fourth is eliminated via urine.^26^ These pharmacokinetic properties highlight the drug's potential for sustained therapeutic effects and suggest a need for close monitoring of hepatic function and drug interactions during therapeutic usage, which exerts its specific action through various receptors such as VEGF receptors (VEGFR), fibroblast growth factor receptors (FGFRs), platelet‐derived growth factor receptor‐alpha (PDGFR‐α), KIT, and RET, by binding to them, thereby preventing phosphorylation of their downstream targets and concomitantly suppressing the reaction cascade of aberrant cell proliferation.^25^ VEGF and FGF signaling play crucial roles in sustained angiogenesis, invasion, and metastasis, which are hallmarks of carcinogenesis. Notably, lenvatinib's inhibition of VEGF and FGF signaling has anti‐angiogenic and immunomodulatory effects (Fig. 5). Lenvatinib leads to a conversion of the inherently immunosuppressive milieu of the tumor microenvironment into a state that actively stimulates the immune response.^27^, ^28^, ^29^, ^30^

Sorafenib exerts its pro‐apoptotic, anti‐angiogenic, and antitumor properties mainly through VEGFR.^31^ It has no effect against FGFR1‐4, like lenvatinib.

### Interpretation of findings

Our study showed benefits in terms of OS among patients in the lenvatinib group compared to those in the sorafenib group. The result obtained agrees with the OS outcomes in a study by Rimini *et al*.^19^ In contrast, comparable outcomes in terms of OS were obtained in some studies.^12^, ^13^, ^22^, ^32^, ^33^ Facciorusso *et al*.^34^ conducted a previous meta‐analysis including five studies with a total of 1481 patients, which revealed no significant variation in OS between the two groups. However, unlike this study, Facciorusso *et al*.'s analysis considered only a limited number of studies with relatively small sample sizes, which may have resulted in uneven outcomes.^34^ According to Sasaki *et al*. patients who received lenvatinib at a higher relative dose intensity (RDI) (>67%) at 8 weeks had substantially higher OS than those who had a lower RDI (<67%).^35^

PFS is the period between the initiation of therapy or Phase III randomization to the onset of disease or death.^36^ Although PFS and ORR have been proposed as potential alternatives to OS for emerging therapies in cancer studies, OS remains an objective primary endpoint to gauge these therapies. Before the introduction of more effective medications, both PFS and ORR might have been evaluated to see whether survival advantages were being consistently reflected.^37^ This meta‐analysis demonstrated a significantly improved PFS with the use of lenvatinib compared to sorafenib in HCC patients. These findings are consistent with previous studies conducted by Rimini *et al*.^19^ Burgio *et al*.^10^ Tomonari *et al*.^22^ and Kim *et al*.^33^ Llovet *et al*. found that PFS is closely associated with OS at the trial level and that PFS with an HR threshold of ≤0.6 is a strong predictor of a noteworthy enhancement in OS.^38^ Hatanaka *et al*. reported that PFS was shorter in patients with extrahepatic spread than those without extrahepatic spread.^36^ Another retrospective investigation found that the pretreatment variables CP‐5A and a tumor size of 40 mm were relevant and that the prevalence of thyroid dysfunction and appetite loss was linked to a poorer PFS.^39^ In addition, this study also highlights a remarkable improvement in DCR with lenvatinib in comparison to sorafenib, similar to the results obtained by Kuo *et al*. (62.3% *vs* 48.6%, *P* = 0.029),^18^ Lee *et al*.^12^ Rimini *et al*. (*P* = 0.002),^19^ Nakano *et al*. (69% *vs* 46%; *P* < 0.0001),^14^ and Kuzuya *et al*. (92.3% *vs* 35.7%; *P* = 0.0008).^21^ All the results obtained thus far provide a better therapeutic advantage to lenvatinib than sorafenib regarding efficacy. The study conducted by Kim *et al*. was not incorporated into our meta‐analysis because of the absence of HRs for the key endpoints such as OS and PFS.^20^ Similarly, the study by Lee *et al*. in 2022 was excluded from our analysis because it provided only median OS data presented in months, without accompanying HRs. Furthermore, their study did not furnish relevant information pertaining to PFS, ORR, and DCR.^12^ These exclusions were made to maintain the robustness and accuracy of our analysis according to stringent scientific standards.

### Limitation

The results of this meta‐analysis should be interpreted in the context of the following limitations. First, the majority of studies were observational, with just one RCT included; therefore, the risk of confounding bias cannot be ruled out. We could not perform regression and subgroup analyses because of the lack of data on the patient's baseline characteristics. Studies with a single arm or studies without outcomes of interest have not been considered to enable us to follow the inclusion criteria strictly.

## Conclusion

Our study shows that lenvatinib is superior to sorafenib in regard to OS and PFS in patients with advanced HCC.

## Supporting information


**Table S1.** Newcastle–Ottawa scale for quality and bias assessment of observational studies.
**Figure S1.** The Preferred Reporting Items for Systematic Reviews and Meta‐Analyses (PRISMA) flow diagram.
**Figure S2.** Subgroup analysis based on sample size for overall survival.
**Figure S3.** Subgroup analysis based on sample size for progression‐free survival.
**Figure S4.** Subgroup analysis based on sample size for time to progression.
**Figure S5.** Subgroup analysis based on sample size for objective response rate.
**Figure S6.** Subgroup analysis based on sample size for disease control rate.
**Figure S7.** Funnel plot for primary outcome.
