# Supplementary material for: Efficacy of lenvatinib versus sorafenib in the primary treatment of advanced hepatocellular carcinoma: A meta‐analysis
Source: JGH Open. 2023 Dec 13;7(12):832–40. doi: 10.1002/jgh3.12999 (PMC10757498; doi:10.1002/jgh3.12999)
Supplement: Supplementary file 1 — Table S1. Newcastle–Ottawa scale for quality and bias assessment of observational studies. Figure S1. The Preferred Reporting Items for Systematic Reviews and Meta‐Analyses (PRISMA) flow diagram. Figure S2. Subgroup analysis based on sample size for overall survival. Figure S3. Subgroup analysis based on sample size for progression‐free survival. Figure S4. Subgroup analysis based on sample size for time to progression. Figure S5. Subgroup analysis based on sample size for objective response rate. Figure S6. Subgroup analysis based on sample size for disease control rate. Figure S7. Funnel plot for primary outcome. [file JGH3-7-832-s001.docx]

**Supplementary Table 1:** Newcastle-Ottawa scale for quality assessment and bias assessment of observational studies.

**Supplementary Figure 1**: The Preferred Reporting Items for Systematic Reviews and Meta-Analyses (PRISMA) flow diagram

**Supplementary Figure 2:** Subgroup analysis based on sample size for overall survival.

**Supplementary Figure 3:** Subgroup analysis based on sample size for progression free survival.

**Supplementary Figure 4:** Subgroup analysis based on sample size for Time to progression.

**Supplementary Figure 5:** Subgroup analysis based on sample size for Objective response rate.

**Supplementary Figure 6:** Subgroup analysis based on sample size for disease control rate.

**Supplementary Figure 7:** Funnel plot for Primary outcome

| **Study** | Selection | Comparability | Outcome | Total |
| --- | --- | --- | --- | --- |
| **Kuzuya et al.** | ** | ** | *** | 7/9 |
| **Nakano et al.** | **** | ** | *** | 9/9 |
| **Tomonari et al.** | *** | ** | *** | 8/9 |
| **Choi et al.** | ** | ** | *** | 7/9 |
| **Burgio et al.** | *** | ** | ** | 7/9 |
| **Rimini et al.** | *** | ** | ** | 8/9 |
| **Lee et al.** | *** | ** | ** | 8/9 |
| **Kuo et al.** | *** | ** | *** | 8/9 |
| **Casadei et al.** | *** | ** | *** | 8/9 |
| **Park et al.** | ** | ** | *** | 7/9 |
| **Fukushima et al.** | ** | ** | *** | 7/9 |
| **Terashima et al.** | *** | ** | ** | 7/9 |

**Supplementary Table 1:** Newcastle-Ottawa scale for quality assessment and bias assessment of observational studies.

**Supplementary Table 2.** Cochrane Collaboration’s tool for assessing the risk of bias in randomized controlled trials

| **Trial Name** | Sequence generation | Allocations concealment | Performance Bias | Detection Bias | Attrition Bias | Reporting Bias |
| --- | --- | --- | --- | --- | --- | --- |
| **Kudo et al.** | LOW | LOW | LOW | LOW | LOW | LOW |

**
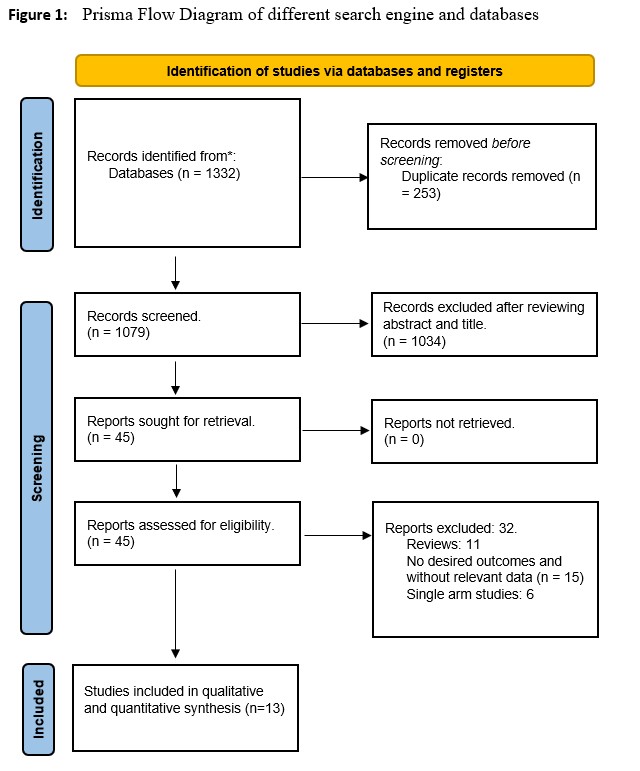
**

**Supplementary Figure 1**: The Preferred Reporting Items for Systematic Reviews and Meta-Analyses (PRISMA) flow diagram

**
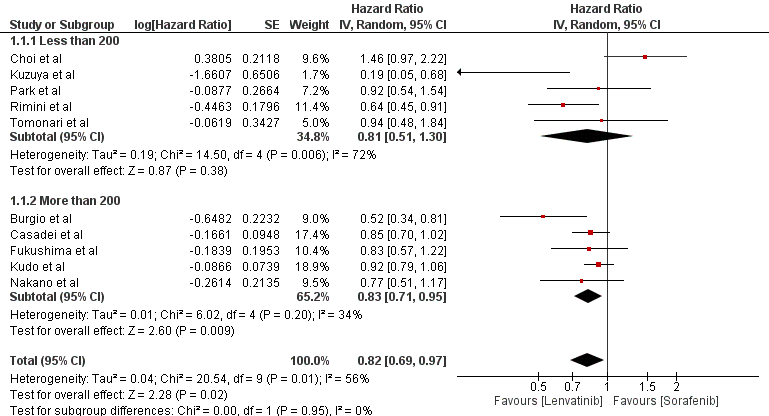
**

**Supplementary Figure 2:** Subgroup analysis based on sample size for overall survival.


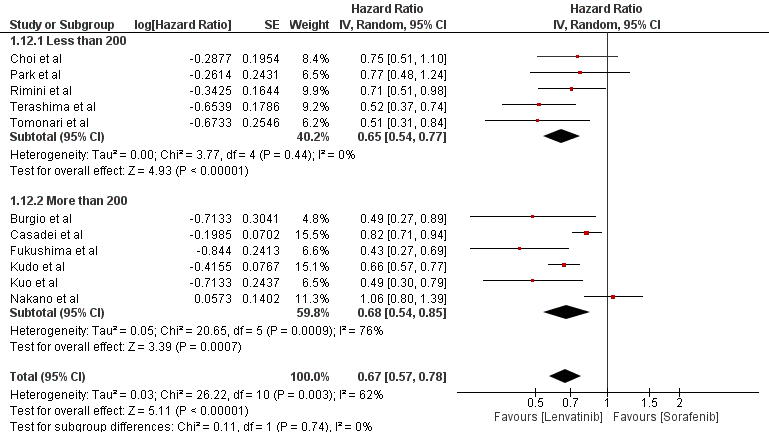


**Supplementary Figure 3:** Subgroup analysis based on sample size for progression free survival.


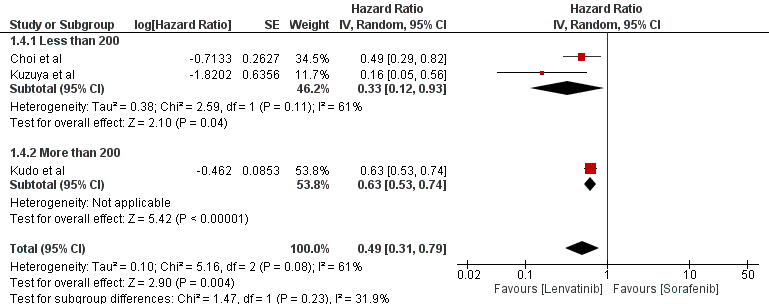


**Supplementary Figure 4:** Subgroup analysis based on sample size for Time to progression.


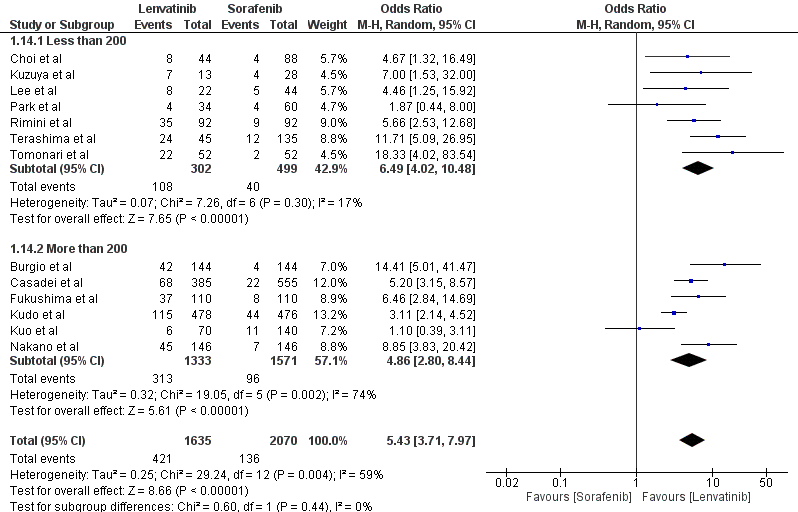


**Supplementary Figure 5:** Subgroup analysis based on sample size for Objective response rate.


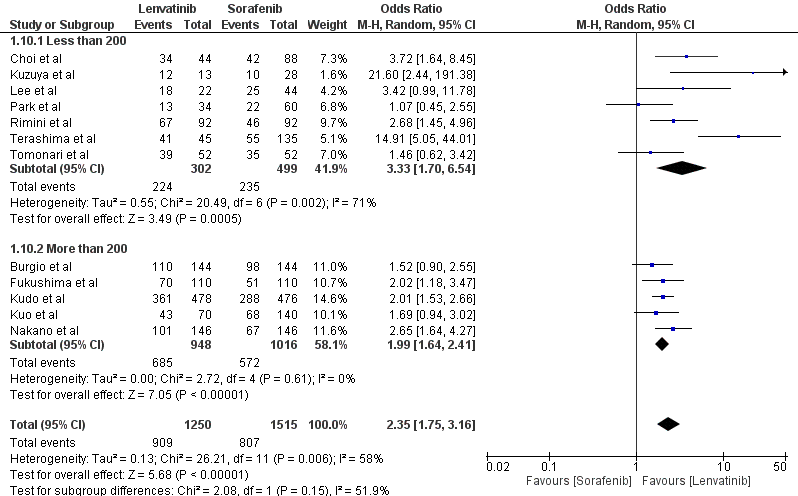


**Supplementary Figure 6:** Subgroup analysis based on sample size for disease control rate.

**
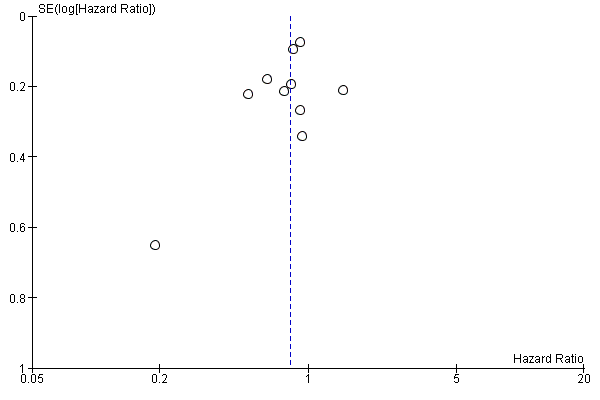
**

**Supplementary Figure 7:** Funnel plot for primary outcome: Overall survival
